# A Conserved CXXC Motif in CD3ε Is Critical for T Cell Development and TCR Signaling

**DOI:** 10.1371/journal.pbio.1000253

**Published:** 2009-12-01

**Authors:** Yibing Wang, Dean Becker, Tibor Vass, Janice White, Philippa Marrack, John W. Kappler

**Affiliations:** 1 Integrated Department of Immunology, National Jewish Health, Denver, Colorado, United States of America; 2 Howard Hughes Medical Institute, National Jewish Health, Denver, Colorado, United States of America; 3 Department of Biochemistry and Molecular Genetics, University of Colorado at Denver and Health Sciences Center, Aurora, Colorado, United States of America; 4 Program in Biomolecular Structure, University of Colorado at Denver and Health Sciences Center, Aurora, Colorado, United States of America; Harvard Medical School, United States of America

## Abstract

Virtually all T cell development and functions depend on its antigen receptor. The T cell receptor (TCR) is a multi-protein complex, comprised of a ligand binding module and a signal transmission module. The signal transmission module includes proteins from CD3 family (CD3ε, CD3δ, CD3γ) as well as the ζ chain protein. The CD3 proteins have a short extracellular stalk connecting their Ig-like domains to their transmembrane regions. These stalks contain a highly evolutionarily conserved CXXC motif, whose function is unknown. To understand the function of these two conserved cysteines, we generated mice that lacked endogenous CD3ε but expressed a transgenic CD3ε molecule in which these cysteines were mutated to serines. Our results show that the mutated CD3ε could incorporate into the TCR complex and rescue surface TCR expression in CD3ε null mice. In the CD3ε mutant mice, all stages of T cell development and activation that are TCR-dependent were impaired, but not eliminated, including activation of mature naïve T cells with the MHCII presented superantigen, staphylococcal enterotoxin B, or with a strong TCR cross-linking antibody specific for either TCR-Cβ or CD3ε. These results argue against a simple aggregation model for TCR signaling and suggest that the stalks of the CD3 proteins may be critical in transmitting part of the activation signal directly through the membrane.

## Abstract 2

Structural integrity of the extracellular membrane-proximal stalk region of CD3ε is required for efficient signaling by the T cell antigen receptor complex. The results in this article suggest that receptor aggregation may not be sufficient for a complete T cell receptor signal and that some type of direct allosteric signal may be involved.

## Author Summary

The T cells of the immune system have surface receptors that detect unique features (called antigens) of foreign invaders such as viruses, bacteria and toxins. An encounter between an antigen and the T cell receptor sets off a chain of events that activates the T cell to proliferate and thus call to action the various arms of the immune response that ultimately eliminate the invader. A set of proteins, called CD3, associates with the T cell receptor, spanning the cell membrane. Their function is to deliver a signal to the inside of T cell that its receptor has encountered antigen on the outside of the cell. Two general ideas have been proposed to explain how the CD3 proteins accomplish this: That the engagement of the T cell receptor outside the cell directly causes a change in conformation in the intracellular portion of the associated CD3 proteins that is recognized by the intracellular signaling machinery; and that engagement of the T cell receptor causes clustering of multiple receptor and CD3 proteins such that interactions among the cytoplasmic portions of the many CD3 proteins now attract other proteins to start the chain of intercellular signaling. These two ideas are not mutually exclusive. We show here that mutations in a highly conserved extracellular portion of one of the CD3 proteins can impair the transmission of the activation signal without preventing receptor clustering. These results suggest that direct transmission of a conformational change across the membrane may constitute part of the CD3-mediated activation signal.

## Introduction

The vertebrate immune system is an evolutionarily selected, highly developed defense system that includes an innate component that can be traced back to unicellular organisms and an adaptive or antigen-specific immune response unique to vertebrates. The ability of the antigen-specific cells of the adaptive response to expand and give rise to immune memory can rid the body of infection and afford life-long resistance to re-infection. A major component of the adaptive immune system is the set of T cells that bear αβ antigen receptors (TCRs). Virtually all aspects of the development and function of these T cells involve signaling cascades initiated from their TCR complexes. These include the initial expansion of thymic TCRβ^+^ pre-T cells, subsequent self-MHC driven thymic positive and negative selection, self-MHC dependent peripheral homeostasis, and of course, the response of mature T cells to MHC presented foreign antigens.

The αβ TCR complex is composed of two functionally different modules: one for ligand binding and one for signal transmission. The ligand-binding module is composed of two variable polypeptide chains, TCRα and β, which form a covalently linked heterodimer and are responsible for the ligand specificity of the TCR. The signal-transmission module of the TCR complex, however, is composed of invariant polypeptide chains, including CD3ε, CD3γ, CD3δ, and ζ. Among them, CD3ε, CD3γ, and CD3δ form non-covalently linked CD3εγ and CD3εδ heterodimers, whereas ζ forms a covalently linked ζζ homodimer (reviewed in [1]). Surface expression of the TCR complex requires a fully assembled set of the complex subunits (reviewed in [2]). Assembly begins with the formation, in the endoplasmic reticulum, of CD3εδ and CD3εγ heterodimers [3]. These then associate with TCRα and TCRβ, respectively, to generate intermediate complexes [3],[4]. The ζζ homodimer is the last subunit to join, and upon its incorporation, the whole TCR complex is transported to the plasma membrane [5],[6].

While the pre-TCR is thought to signal constitutively (reviewed in [7]), at all subsequent developmental stages, engagement of the TCR by an MHC ligand is required for signaling. There is still no consensus on the details of how this signaling is initiated. Some evidence supports a mechanism in which ligand dependent TCR aggregation allows kinases associated with the cytoplasmic tails of CD3 and co-receptors to cross-phosphorylate their targets [8]–[11]. Other experiments have suggested that individual TCRs, when engaged, can directly transmit a signal through the membrane, which open sites in the cytoplasmic regions of the CD3 molecules that allow binding of other proteins involved in signal propagation [12]–[17]. These two ideas are not mutually exclusive [18],[19].

**Figure 1:**
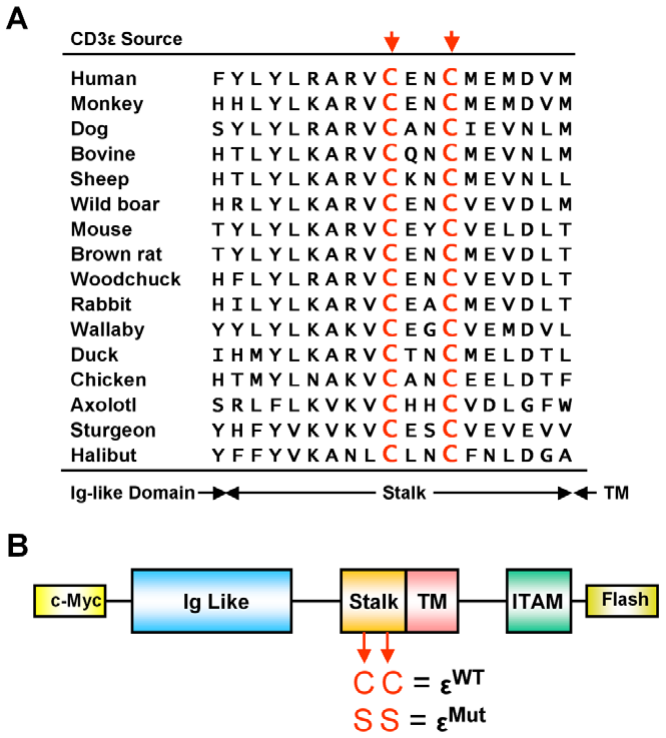
Two cysteines in the stalk region of CD3ε are highly conserved and form a CXXC motif. (A) Protein sequences of CD3ε from different species are aligned using the Clustal W program (http://www.ebi.ac.uk/clustalw/). The hypothetical boundaries of the stalk region are defined based on the mouse CD3ε protein. The two conserved cysteines in the stalk region are colored red and marked with the arrowheads. (B) A schematic view of constructed CD3ε proteins. Both of the non-mutated (ε^WT^) and C-to-S mutated (ε^Mut^) murine CD3ε primary sequences have a c-Myc and flash tag sequence on each end. cDNA fragments encoding these sequences were cloned into a human CD2 minigene cassette and subsequently generated transgenic mice were bred to endogenous cd3ε null background (see Materials and Methods for details).

A strikingly evolutionarily conserved feature of the CD3 γ, δ, and ε molecules is a pair of cysteines in the short “stalk” connecting their N-terminal extracellular Ig-like domains to their transmembrane regions (Figure 1A). Intermolecular disulfide bonds among these proteins have not usually been seen. Therefore, the chemical status and conserved function of these cysteines in vivo are still under debate, although many favor a structural role via a short intrachain disulfide [20]–[23]. In this study, we compared mice that expressed wild-type (wt) CD3ε to those in which the two cysteines in the CD3ε stalk were replaced with serines. We found that αβTCR surface expression on CD4^+^CD8^+^ (DP) thymocytes and naïve peripheral T cells were equivalent in the CD3ε wt and mutant transgenic mice. However, the mutation caused a profound bottleneck in all TCR dependent processes in T cell development and compromised in the signaling function of the TCR in the naïve peripheral T cells. Our data suggest that TCR aggregation is not sufficient for complete TCR activation and that the conserved CD3 cysteines may play a direct structural role in transmitting the signal that the TCR has engaged ligand across the plasma membrane to the cell interior.

## Results

### Transgenic Mice with T Cells Bearing wt or Mutated CD3ε

Our purpose was to compare the development and activation of T cells whose TCR complexes contained either CD3ε whose cysteines in the stalk region were not mutated (tgε^WT^) or CD3ε with cysteines mutated to serines (tgε^Mut^). As described in Figure 1B and the Materials and Methods, we created transgenic mice bearing constructs encoding ε^WT^ or ε^Mut^ expressed under control of the human CD2 enhancer/promoter. These transgenes were bred into CD3ε null mice (cd3ε^−/−^) so that they would be the only sources of CD3ε for the TCR complex.

### Similar Expression of the αβTCR Complex on tgε^WT^ and tgε^Mut^ T Cells

Initial characterization of the backcrossed transgenic mice confirmed the functionality of our constructs, showing that both tgε^WT^ and tgε^Mut^ could restore the appearance of peripheral T cells in mice lacking endogenous CD3ε. Backcrossed mice expressing the highest surface level of TCR on peripheral T cells were selected for continued breeding and a more detailed analysis. Since previous in vitro experiments had suggested that mutation of the CD3ε CXXC motif could affect the ability of CD3ε to assemble into a complete TCR complex [23], we carefully examined the tgε^WT^ and tgε^Mut^ mice for the surface level of the αβTCR on various subpopulations of T cells. The results are shown in Table 1. There was no difference in αβTCR expression on the DP thymocytes comparing the tgε^WT^ and tgε^Mut^ mice. There was some reduction (∼25%–50%) in αβTCR expression on total mature peripheral T cells in the tgε^Mut^ mice compared to the tgε^WT^ mice, especially on CD4^+^ T cells. Since there was virtually no difference when we examined only peripheral T cells with a naïve phenotype, we eventually attributed this lower surface TCR level to the large proportion of the T cells that homeostatically expanded in the tgε^Mut^ mice, which obtained a memory-like phenotype (see below).

**Table 1:** Levels of TCR and CD3εγ on the surface of immature or mature T cells from transgenic and C57BL/6 mice.

	DP Thymocytesa,b	Naïve CD4^+^ T Cells^b^	Naïve CD8^+^ T Cells^b^	Total CD4^+^ T Cellsb,c		Total CD8^+^ T Cellsb,c	
	Anti TCR-Cβ (H57–597)^d^	Anti TCR-Cβ (H57–597)^d^	Anti TCR-Cβ (H57–597)^d^	Anti TCR-Cβ (H57–597)^d^	Anti CD3εγ (7D6)^d^	Anti TCR-Cβ (H57–597)^d^	Anti CD3εγ (7D6)^d^
tgε^WT^	100	100	100	100	100	100	100
tgε^Mut^	92.5±4.4	87.5±6.1	91.2±7.9	58.9±13.7	72.4±5.6	72.8±10.3	88.4±15.0
C57BL/6	98.8	ND^e^	ND^e^	133.1±16.6	178.1	184.4±29.6	212.2

a DP thymocytes were suspended and cultured in medium at 37°C for 4 h before analysis.

b Values are the geometric average of the mean fluorescence intensity (expressed as the percent of the value obtained with tgε^WT^ cells) ± SD.

c Total CD4^+^ or CD8^+^ T cells were from peripheral spleen and lymph nodes.

d mAb used for flow cytometry.

e “ND” refers to “not done.”

In our experiments tgε^Mut^ transgenic mice were compared not only to tgε^WT^ transgenic mice but also to normal C57BL/6 mice and to the endogenous CD3ε heterozygous littermates of the two transgenic lines: tgε^WT^ cd3ε^+/−^ and tgε^Mut^ cd3ε^+/−^. At any stage of T cell development, we found no significant phenotypic differences between the tgε^WT^ mice and any of the other control mice containing at least one functional copy of the endogenous CD3ε gene, despite the fact that the TCR level on tgε^WT^ peripheral T cell was about 50% lower than that on the normal C57BL/6 T cells (Table 1). These results indicated that the presence of a wt CD3ε molecule in any form was sufficient for normal T cell development and function. Therefore, for simplicity of presentation, in most of the experiments presented below, we combined the data for the four types of positive control mice. The actual numbers of the different types of control mice for any particular experiment are listed in the figure legends.

### Transgenic tgε^Mut^ Mice Have Defects in T Cell Development in the Thymus

**Figure 2:**
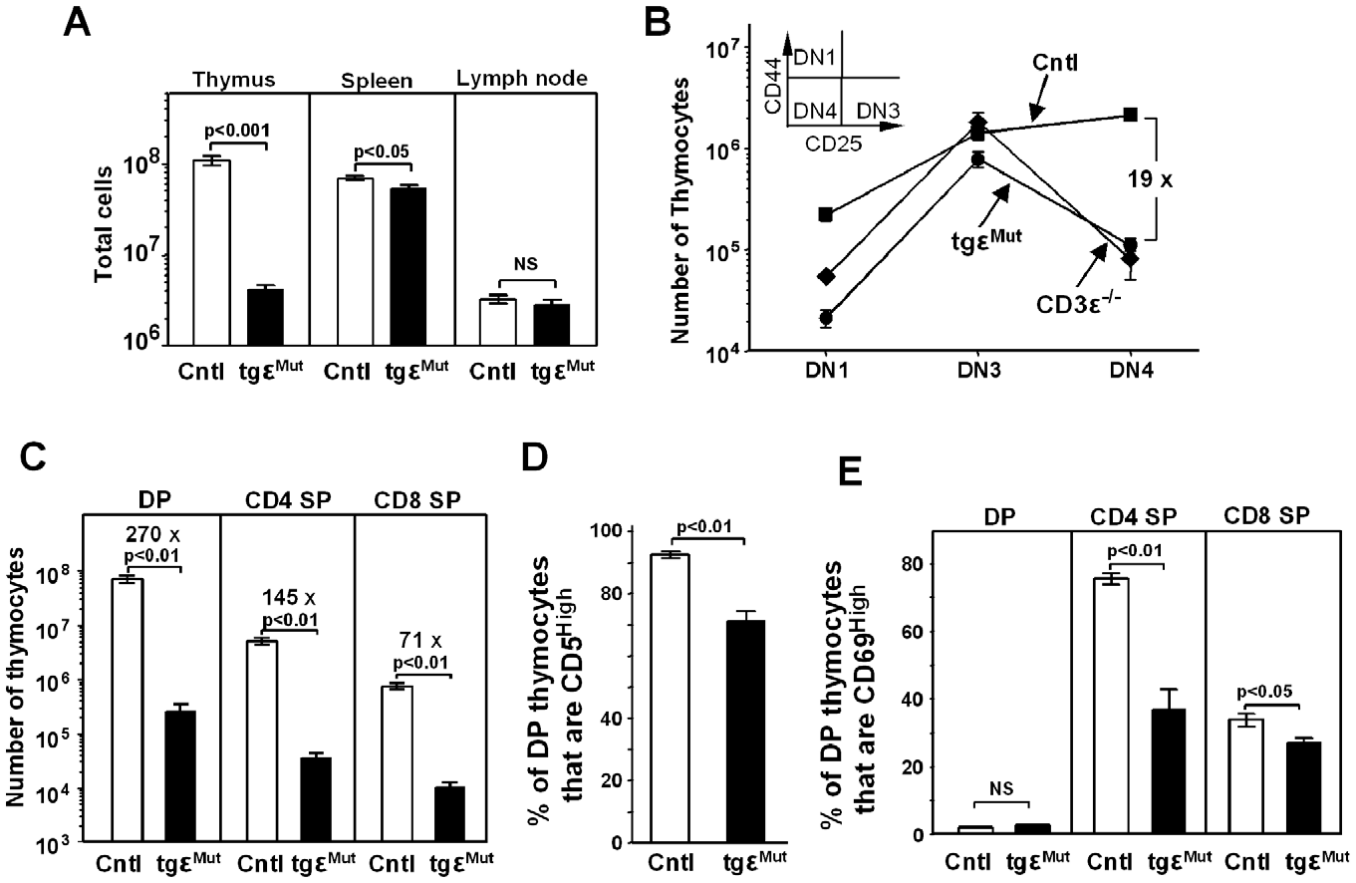
Transgenic mice with cysteine mutations have defects in T cell development in the thymus. (A) Total cell counts in various organs in the combined wt control (Cntl, open bars) versus the mutant transgenic mice (tgε^Mut^, filled bars). Cell counts were obtained after the lysis of red blood cells. Data from lymph nodes represent cell counts from inguinal, brachial, and axillary lymph nodes combined and are shown as the number per lymph node. Data shown are for 8 tgε^Mut^ mice and 13 wt control mice (4 BL6, 1 tgε^WT^cd3ε^+/−^, 5 tgε^Mut^cd3ε^+/−^, and 3 tgε^WT^). (B) Total cell counts in the thymus that were at the DN1, DN3, or DN4 stage. The stages are defined by CD44 and CD25 costaining, with a schematic view shown in the upper-left corner of the graph. Data shown are for 8 tgε^Mut^ mice (circles) and 13 wt control mice (squares) as in (A) and 2 cd3ε^−/−^ mice (diamonds). (C) Number of thymocytes that were at the double positive (DP) or single positive stages (CD4 SP and CD8 SP) in control (open bars) versus tgε^Mut^ (filled bars) mice. The numbers of mice examined are as in (A). (D) The percentage of CD5^hi^ cells at the double positive stage of T cell development in the thymuses of control (open bars) versus tgε^Mut^ (filled bars) mice. The numbers of mice examined are as in (A). (E) The percentage of CD69^hi^ cells at the double positive or single positive stages of T cell development in the thymuses of control (open bars) versus tgε^Mut^ (filled bars) mice. Data shown are for 4 tgε^Mut^ mice and 8 wt control (cntl) mice (3 BL6, 1 tgε^WT^cd3ε^+/−^, 1 tgε^Mut^cd3ε^+/−^, and 3 tgε^WT^). For (B–E) cells from the thymus were costained with multiple antibodies against surface proteins (CD4, CD8, B220, CD44, CD25, CD5, and CD69). To avoid including non-T lineage cells and minimize nonspecific staining, only live B220^−^ cells were analyzed. The mean of the group and the SEM are indicated. *p* values were obtained from *t* tests. In some cases, fold decreases are indicated.

To study the potential effects of the ε^Mut^ on the development of T cells, we first looked at the cellularity in the relevant organs, that is, thymus, spleen, and lymph nodes. As shown in Figure 2A, the tgε^Mut^ thymuses had only about 4% of the number of cells present in the control thymuses. This indicated a bottleneck in T cell development. CD3ε is required for thymic T cell development and CD3ε null mice do not have T cells beyond the triple negative stage (CD4^−^CD8^−^CD44^−^CD25^+^) because of the lack of pre-TCR signals [24],[25]. Thus, we suspected that mutating the conserved cysteines in the CD3ε stalk might also affect T cell development beginning at an early stage.

To test this hypothesis, we examined the number of thymocytes at various CD4^−^CD8^−^ (DN) stages. As shown in Figure 2B, the thymocytes from control mice increased in number from the DN1 to DN4 stage. Those from CD3ε null mice, however, were blocked at the DN3 stage. The poor transition of tgε^Mut^ thymocytes from the DN3 to DN4 stage suggested a defect in pre-TCR signals. This bottleneck at the DN3 stage was not as drastic for the tgε^Mut^ mice as it was for CD3ε null mice. The block at DN3 is nearly complete for CD3ε null thymocytes since virtually no cells make it to the subsequent DP or CD4^+^ or CD8^+^ ( single positive, SP) stages [24]. However, while the number of DP and SP thymocytes was greatly reduced in the tgε^Mut^ compared to control mice, a significant number of tgε^Mut^ thymocytes were present at the DP stage as well as at the SP stages (Figure 2C).

The surface phenotype of the tgε^Mut^ DP and SP thymocytes indicated there was also a bottleneck in the TCR-driven DP to SP transition. For example, up-regulation of CD5 expression on DP thymocytes is thought to be an indicator of the intensity of interactions between TCRs and self MHC-peptides during selection [26]. As shown in Figure 2D, nearly all of the DP cells in the control mice had elevated expression of CD5. On the other hand, significantly fewer DP cells in the tgε^Mut^ mice had up-regulated CD5 expression. This indicated that fewer tgε^Mut^ DP cells received sufficient signals through the interaction between their TCRs and self MHC-peptides.

CD69 has been reported to be transiently expressed on thymocytes that are undergoing or have just finished the process of TCR-mediated positive selection [27]–[29]. Thus, its expression on SP cells is a useful marker for recently selected cells. As shown in Figure 2E, CD8^+^ SP and, more markedly, CD4^+^ SP thymocytes from tgε^Mut^ mice had significantly fewer cells that were CD69^hi^ than their counterparts in control mice. These results, coupled with the CD5 data, indicated that positive selection in the mutant mice was impaired.

Thus, mutation of the conserved cysteines in the CD3ε stalk region had a profound impact on thymocyte development. It not only reduced output of cells during the DN3 to DN4 transition, indicating a defect in pre-TCR signals, but also introduced a defect in TCR-mediated selection during the DP and SP stages. These results explained the reduced cellularity in the tgε^Mut^ thymuses and predicted a much reduced rate of thymic output to the periphery.

### Thymic Output Is Reduced and Many Peripheral T Cells Homeostatically Proliferate in tgε^Mut^ Mice

**Figure 3:**
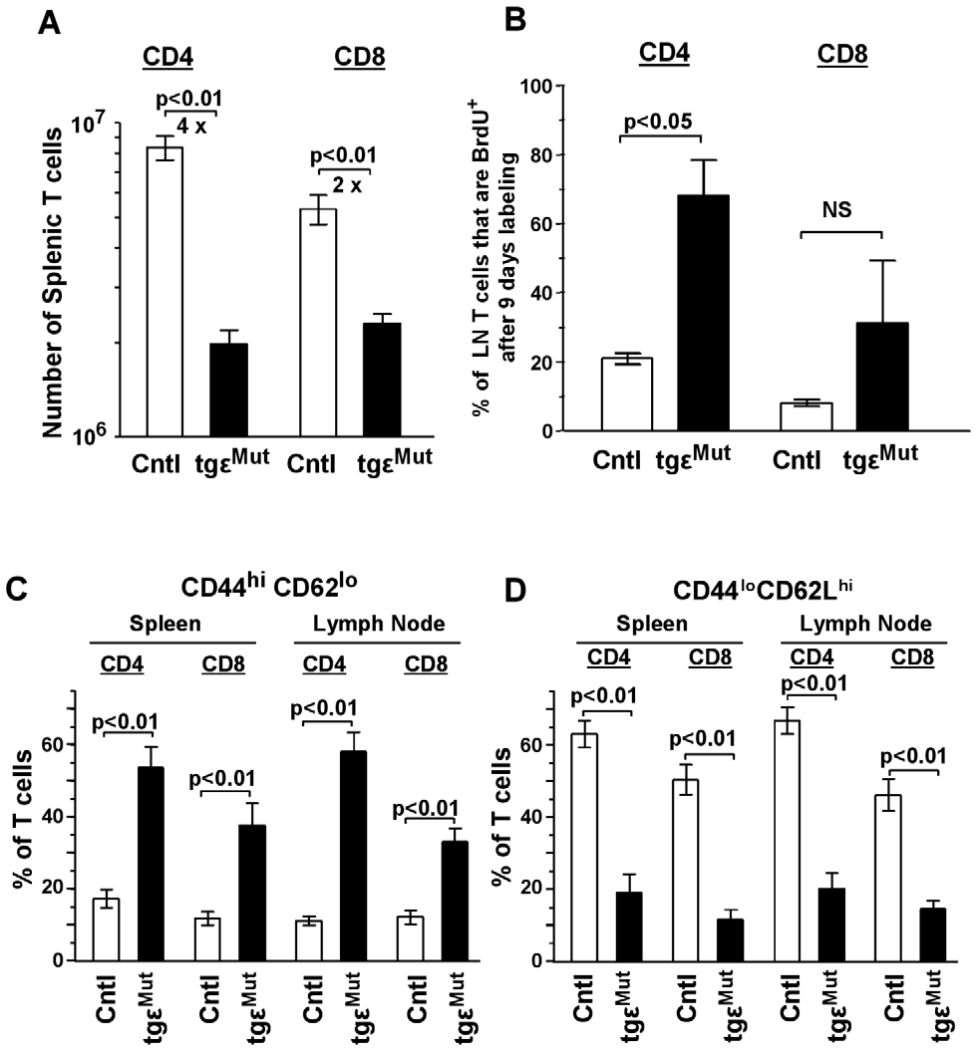
The numbers of mature T cells from transgenic mice bearing mutated CD3ε were decreased, and they were actively proliferating in a homeostatic manner. In all panels data for control mice are open bars and for tgε^Mut^ mice are filled bars. (A) The cell counts of mature CD4^+^ or CD8^+^ T cells in the spleen. Fold decreases for the two populations are indicated. Data shown are for 8 tgε^Mut^ mice and 13 wt control mice (4 BL6, 1 tgε^WT^cd3ε^+/−^, 5 tgε^Mut^cd3ε^+/−^, and 3 tgε^WT^). (B) The incorporation of BrdU into the mature T cells. Mice were given drinking water containing 0.8 mg/ml BrdU for 9 consecutive days before sacrifice. Data shown are three mice for each group. (C, D) The percentage of CD44^hi^CD62L^lo^ or CD44^lo^CD62L^hi^ T cells in the CD4^+^ or CD8^+^ population of spleen and lymph nodes. The numbers of mice examined are as in (A). Spleen or pooled lymph node (inguinal, brachial, and axillary) cells were costained with antibodies against B220, CD4, CD8, CD44, CD62L, as well as BrdU in the case of (B). The percentages were obtained from flow cytometry data by gating on live B220^−^CD4^+^ or B220^−^CD8^+^ cells. Data are shown as the mean ± SEM. *p* values were obtained from *t* tests.

In the spleen, the tgε^Mut^ mice had fewer CD4^+^ and CD8^+^ T cells than all the wt controls (Figure 3A), but the extent of the decrease (about 4-fold less for the CD4^+^ and 2-fold less for the CD8^+^ T cells) was not as severe as in the thymus (Figure 2C). The most likely explanation for this difference between the thymus and periphery was the gradual accumulation of peripheral T cells in the tgε^Mut^ mice and expansion of their number as a result of lymphopenia driven homeostasis.

To test this idea, we first examined the status of these T cells in terms of proliferation. Tgε^Mut^ and control mice were given bromodeoxyuridine (BrdU) (0.8 mg/ml) in their drinking water for 9 consecutive days after which BrdU incorporation into CD4^+^ and CD8^+^ T cells was assessed by flow cytometry (Figure 3B). Only ∼15%–20% of the control T cells incorporated BrdU, consistent with the observations in normal mice in a previous study [30]. In the tgε^Mut^ mice, on the other hand, more T cells incorporated BrdU, especially in the CD4^+^ population. This indicated that more peripheral T cells in the tgε^Mut^ mice were actively proliferating, probably due to homeostatic expansion.

We then examined the activation status of these T cells in terms of surface markers, because it has been reported that naïve T cells acquire a memory-like phenotype through lymphopenia-driven proliferation [31]–[34]. As showed in Figure 3C and 3D, the control mice had a substantial population of peripheral T cells with a naïve phenotype (CD44^lo^CD62L^hi^) and few T cells with a memory-like phenotype (CD44^hi^CD62L^lo^). The opposite was seen in the tgε^Mut^ mice. Some T cells remained naïve, but many had acquired a memory-like phenotype. Thus, the proliferative status and surface phenotype of the peripheral T cells in the tgε^Mut^ mice were consistent with the presence of T cells that had gone lymphopenia-induced homeostatic expansion. However, interestingly, this expansion did not result in the full recovery of peripheral T cell numbers in the tgε^Mut^ mice (Figure 3A). Since TCR engagement by self-MHC has been implicated in maintenance of the naïve T cell population [35],[36], it is possible that this TCR dependent process is also impaired somewhat by the mutations in CD3ε.

While homeostatic expansion most likely accounted for some of the size of the peripheral CD4^+^ and CD8^+^ T cell population in the tgε^Mut^ mice, these cells were not oligoclonal. When tested with a set of anti-Vα and anti-Vβ antibodies, all V elements were present in the population, although the exact usage of particular Vαs or Vβs was somewhat skewed (Figure S1). It is possible that this is a consequence of combined effects by CD3ε mutations on both thymic output and homeostatic expansion.

### The Cysteine Mutations Affect the CD4^+^ and CD8^+^ Population Unequally

**Figure 4:**
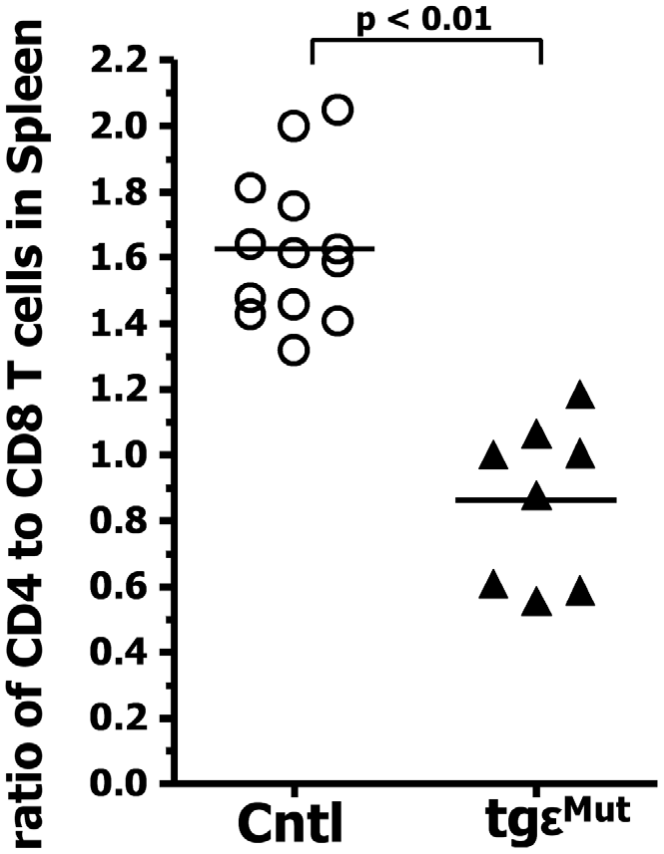
The cysteine mutations affected the CD4^+^ and CD8^+^ population unequally. Spleen cells from control (4 BL6, 1 tgε^WT^cd3ε^+/−^, 5 tgε^Mut^cd3ε^+/−^, and 3 tgε^WT^, open circles) or tgε^Mut^ (filled diamonds) mice were costained using anti-B220, anti-CD4, and anti-CD8 antibodies. The CD4 to CD8 ratio was calculated based on live B220^−^CD4^+^ or the B220^−^CD8^+^ cells. *p* values were obtained using *t* tests.

In the tgε^Mut^ mice, we noticed that the impact of the cysteine mutations was more profound for the CD4^+^ T cell population than for the CD8^+^ T cell population. This difference first appeared in the thymus at the SP stage where the reduction in cell numbers and the poor up-regulation of CD69 was much more striking for CD4^+^ than for CD8^+^ SP cells (Figure 2C, E). It was also true for peripheral T cells (Figure 3A and Figure 4). While, as mentioned above, the overall reduction in peripheral SP T cells in the tgε^Mut^ mice was not as dramatic as in the thymus, the overall effect was still greater for CD4^+^ than for CD8^+^ cells, leading to a significant decrease in the CD4/CD8 ratio. This is probably partially due to the unequal output of CD4^+^ and CD8^+^ T cells from the thymus, but also might indicate a differential effect of the CD3ε mutations on TCR driven homeostatic expansion of the two populations.

### TCR Signaling Is Compromised in Peripheral T Cells from tgε^Mut^ Mice

**Figure 5:**
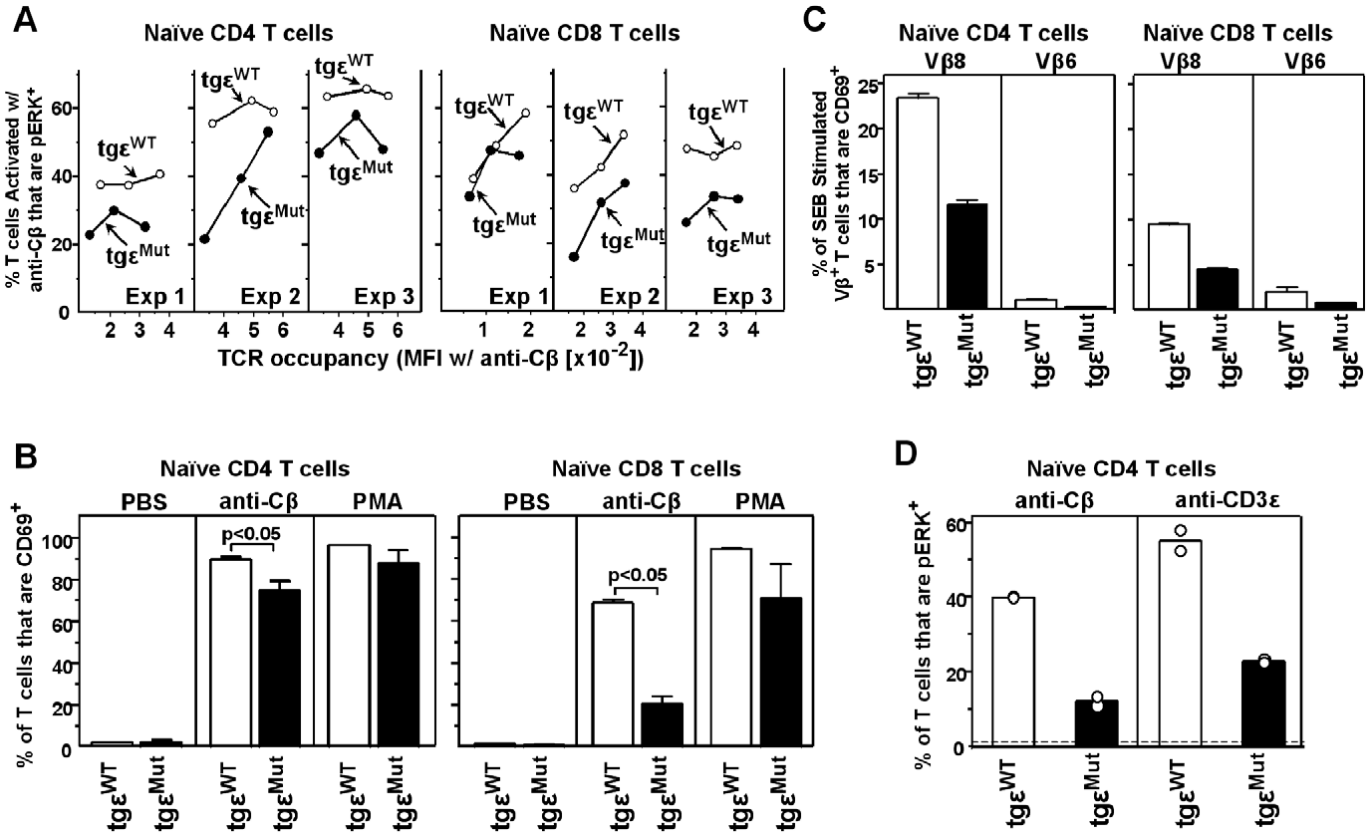
TCR signaling is compromised in the naïve peripheral T cells from tgε^Mut^ mice. (A) ERK1/2 phosphorylation versus TCR occupancy in naïve CD4^+^ or naïve CD8^+^ T cells. As described in Materials and Methods, T cells from spleen and lymph nodes of tgε^WT^ (open circles) or tgε^Mut^ (filled circles) mice were nylon wool-purified and activated for 20 min with various concentrations of anti-Cβ antibody. The percentage of naïve (CD44^lo^) CD4^+^ and CD8^+^ T cells containing pERK was determined. The data are presented as the percent of pERK^+^ cells versus the amount of anti-Cβ antibody bound (geometric mean fluorescence intensities, M.F.I.). The values of pERK^+^ were from the CD44^lo^CD4^+^ or CD44^lo^CD8^+^ populations, hence the “naïve CD4^+^” or “naïve CD8^+^”. In each experiment, cells of each group were pooled from two individual animals. (B) CD69 induction on naïve (CD44^lo^) CD4^+^ or CD8^+^ T cells from tgε^WT^ (open bars) or tgε^Mut^ (filled bars) mice after T cell activation. Nylon wool-purified T cells were stimulated with nothing, plate-bound anti-TCR-Cβ (H57–597) antibody or 20 nM PMA for 12 h. Surface induction of CD69 was assessed by flow cytometry. Data are shown as the mean ± SD for three independent experiments. *p* values are from *t* test. (C) Naïve T cells from the tgε^Mut^ mice respond poorly to superantigen stimulation. Spleen and lymph node cells from tgε^WT^ (open bars) or tgε^Mut^ (filled bars) mice were cultured with SEB (10 µg/ml) for 15 h. The induction of CD69 on CD4^+^ and CD8^+^ naïve T cells bearing either Vβ8 (SEB-reactive) or Vβ6 (SEB-non-reactive) T cells was determined by flow cytometry. The data are expressed as the average increase in cells expressing CD69 (%) over that observed in control cultures that received no SEB. (D) The defect in signaling in the tgε^Mut^ T cells was intrinsic to the CD3ε molecule. Nylon wool-purified T cells from tgε^WT^ (open bars) or tgε^Mut^ (filled bars) mice were stimulated by plate-bound anti-TCR-Cβ or anti-CD3ε antibodies at 37°C for 20 min. After stimulation, CD4^+^ T cells were analyzed for intracellular pERK by flow cytometry. The dash line indicates the control cells that were not stimulated by the antibodies. Data (circles) are from two independent experiments. In each experiment, cells of each group were pooled from two individual animals.

It was important to find out how well the TCRs on the tgε^WT^ and tgε^Mut^ peripheral T cells signaled the cell following TCR engagement. We used two flow cytometric measurements of TCR signaling, cytoplasmic staining for the rapid, transient phosphorylation of ERK1/2 [37] and surface staining for the induction of CD69 [38]. In an initial experiment examining total CD4^+^ or CD8^+^ T cells stimulated with an anti-Cβ mAb, we found that the tgε^Mut^ T cells responded more poorly than the tgε^WT^ T cells in both assays (Figure S2). However, since we subsequently found that the mutant mice had a large population of homeostatically expanded T cells, which had no direct counterpart in the tgε^WT^ mice, we examined the mice again, confining the analyses to those T cells that had a naïve phenotype (Figure 5). After activation by TCR cross-linking with the anti-Cβ mAb for 20 min, fewer CD4^+^ and CD8^+^ naïve T cells from the tgε^Mut^ mice had phosphorylated ERK than from the tgε^WT^ mice (Figure 5A). This is true even at various levels of TCR occupancy by the antibody. Likewise, after activation by TCR cross-linking with the anti-Cβ mAb for 12 h, fewer CD4^+^ and CD8^+^ naïve T cells from the tgε^Mut^ mice had up-regulated CD69 than from the tgε^WT^ mice (Figure 5B). These results established that the TCR complex containing the mutant CD3ε transduced an activation signal into the T cell less efficiently than TCR complexes containing wt CD3ε, despite using a high affinity anti-TCR mAb to cross-link the TCRs on the cell surface.

To see how signaling would be affected by an antigen of much lower affinity presented by MHCII on antigen presenting cells (APCs), we then stimulated the T cells with the bacterial superantigen, SEB. This enterotoxin binds to MHCII on APCs and is recognized by nearly all T cells whose TCRs bear members of the Vβ8 family [39]. The affinities of the enterotoxin superantigens for TCRs bearing the appropriate Vβs fall within the range of affinities seen for conventional peptide-MHC complexes, i.e. KDs in the 1 to 100 uM range [40]. Therefore, spleen and lymph node cells from the tgε^WT^ or tgε^Mut^ mice were incubated with an optimal concentration of SEB for 15 h. The up-regulation of CD69 on the surface of naïve CD4^+^ or CD8^+^ T cells was assessed (Figure 5C). As seen with the anti-Cβ mAb, fewer naïve T cells from the tgε^Mut^ mice than from the tgε^WT^ mice up-regulated CD69 in response to SEB. As a negative control, we examined in the same cultures SEB-nonreactive T cells bearing Vβ6. In this case very few cells from either mouse had up-regulated CD69. Thus, whether we engaged the TCR by a high affinity anti-Cβ mAb or a low affinity MHCII presented superantigen, the mutant TCR complex signaled poorly compared to the wt complex.

Finally, although the TCR containing the mutant CD3ε appeared to be assembled normally, as judged by staining with various anti-TCR and anti-CD3 mAbs (Table 1), one possibility was that a subtle structural defect had disturbed the interface between CD3ε and Cβ and/or Cα, which interrupted the transduction of a “recognition” signal from the TCR αβ to the CD3 complex. To test this idea we used an anti-CD3ε mAb to directly cross-link the receptor complex while bypassing the αβTCR chains. Generation of pERK was compared in naïve CD4^+^ T cells from tgε^WT^ and tgε^Mut^ mice at 20 min after cross-linking the TCR complex with either an anti-Cβ or anti-CD3ε mAb. As showed in Figure 5D, regardless of the cross-linking antibody, fewer T cells from the tgε^Mut^ mice than from the tgε^WT^ mice had pERK. These data strongly suggest that the defect in signaling in the tgε^Mut^ T cells was intrinsic to the CD3ε molecule rather than due to the disruption of its interaction with TCR constant regions.

We conclude from these experiments that TCRs containing the mutant CD3ε, while apparently fully assembled, fail to transduce a fully effective signal across the T cell membrane when engaged via either the αβ or CD3 portion of the TCR complex. This signaling defect is intrinsic to the mutant CD3ε molecule, seen over a wide range of TCR occupancies with various activating signals.

## Discussion

The function of the conserved CXXC motif in the stalk region of the CD3 γ, δ, and ε has been under debate for over a decade. Numerous studies have been performed in vitro to understand the effect on TCR complex assembly of mutation of these cysteines. Some have suggested that the CXXC motif is involved in the dimerization of CD3ε to CD3γ or to CD3δ [21],[23]. Others [20],[22], however, did not find significant impairment of assembly either of CD3 dimers or of the complete TCR complex by mutating those cysteines in CD3ε alone. In preliminary experiments for our studies reported here, we tested our Cys to Ser mutated CD3ε construct for expression in T cell hybridomas that also had endogenous CD3ε. We found that, while the mutant protein could incorporate into the surface TCR complex, it competed poorly against the endogenous CD3ε during the process (Figure S3).

Nevertheless, as shown in Table 1, in our tgε^Mut^ mice, in the absence of competing endogenous CD3ε, the mutated CD3ε incorporated well enough into the TCR complex to rescue surface TCR expression to a level virtually the same as seen with the non-mutated CD3ε. Moreover, the association of mutated CD3ε with CD3γ appeared normal, at least as judged by reactivity with an antibody specific for the associated CD3γ/ε heterodimer (Table 1). Therefore, this transgenic mouse offered us a unique opportunity to study the function of these cysteines in TCR signaling independent of their role in TCR complex assembly. Our results showed that all TCR-dependent developmental stages of T cells were partially impaired in the tgε^Mut^ mice.

The chemical nature of these cysteines in the CD3 molecules is still not known unequivocally. Extracellularly exposed, unprotected cysteines are not common in proteins because they are too reactive to be maintained in a reduced state in the oxidative environment outside the cell. Therefore, several other structural possibilities have been considered for these cysteines. One suggestion was that they were involved in the formation of disulfide bonds to other proteins, e.g. cross-linking CD3 molecules. However, with a few exceptions [23],[41], there is very little evidence to support this idea. Another suggestion has been that the cysteines on adjacent CD3 molecules are involved in tetra-coordination of divalent metal ions, since this is a common motif in nature. However, Borroto et al. [23] and Xu et al. [20] used chelation reagents during TCR complex assembly and did not see any effect, arguing against this idea. In addition, Xu et al. found that mutating single cysteine residues in the CXXC motif led to covalently linked CD3 dimers, suggesting that the two adjacent cysteines were protected very early in the TCR assembly process.

Perhaps the most favored hypothesis is that within the CD3 CXXC motif a short intramolecular disulfide bond forms. Whatever the actual structural form of these CXXC motifs is, our results suggest that they give some important structural conformation to the bundle of stalks of the four CD3 molecules. In the most widely reported structure of the TCR complex, this bundle includes 2ε, 1γ, and 1δ CD3 chains, all of which are required for optimal transmission of a signal from the ligand portion of the TCR complex to the interior of the T cell.

**Figure 6:**
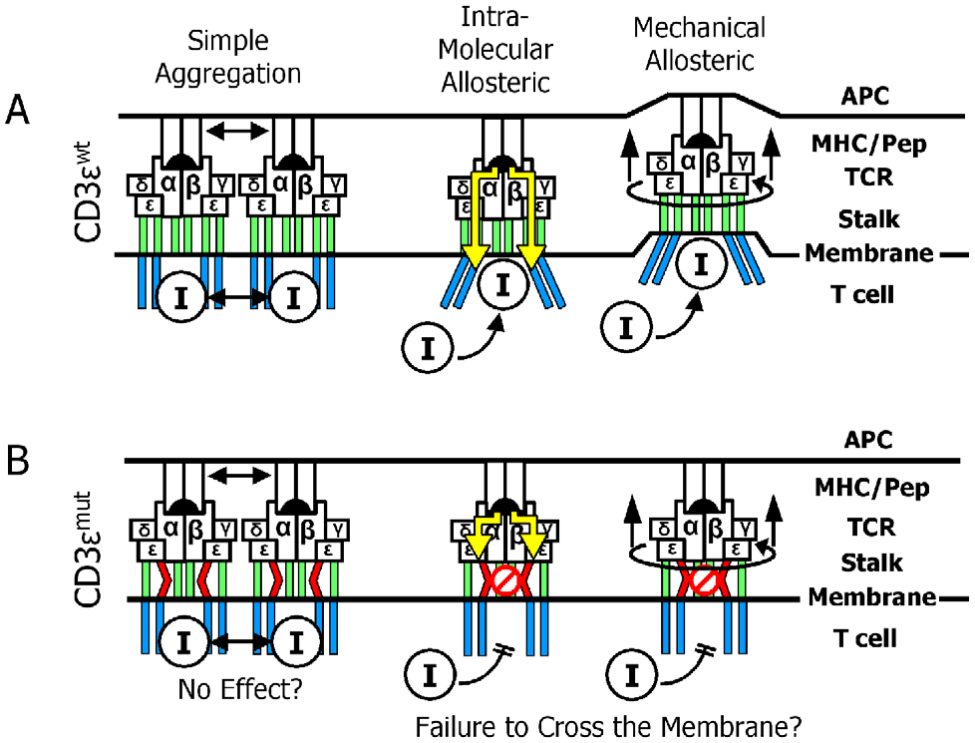
Implications of effects of the CD3ε mutation on models of TCR signal transduction. (A) Schematic representation of proposed models for signal transduction across the T cell membrane to initiate downstream signaling. For simplicity the ζ chain and CD4/CD8 co-receptors are omitted from the diagrams. “I” denotes the critical protein required for initiation of the signaling pathway. See text for discussion. (B) Predicted effect of the CD3ε mutation on the various models. See text for discussion.

The mechanism of TCR complex signaling has been intensely studied over many years. Much has been learned about the downstream pathways activated by TCR engagement, yet a final consensus has not been reached on how engagement of the TCR creates a signal that crosses the cell membrane to initiate the downstream signaling events. Some of the proposed mechanisms are shown schematically in Figure 6A.

The longest standing hypothesis has been a simple aggregation model, originally well laid out by Fewtrell and Metzger [42] to explain IgE Fc receptor signaling and subsequently thought to play a role in many other receptor systems, including the αβTCR. According to this model, in T cells the proteins that initiate the signal are already associated with the cytoplasmic tails of the CD3 proteins, ζ, and the CD4/CD8 co-receptors. However, these proteins do not have access to one another until these molecules coalesce to form clusters, at the interface between the T cell and the APC [10],[11],[43]. Once aggregated, interactions such as cross-phosphorylation among the associated signaling molecules attract other proteins to propagate the signal cascade.

An alternate hypothesis has been that engagement of individual TCRs creates an intrinsic allosteric signal that transverses the T cell membrane to induce a conformational change in the CD3 cytoplasmic tails. At least two types of allostery have been considered. One is the classic intramolecular transmission of conformational change such as seen in the regulation of many enzymes. In this case the binding of the TCR to the MHC/peptide ligand creates a conformational change that is transmitted through the TCR V and C domains to the Ig-like domains, stalks and transmembrane domain of the CD3 molecules leading to a conformational change in the CD3 cytoplasmic tails. Alternately, the allosteric signal could be transmitted mechanically, such as seen with the bacterial aspartate receptor [44]. In this case either translational or torsional forces generated by binding of the APC anchored MHC/peptide to the T cell anchored TCR complex ultimately distorts the CD3 cytoplasmic tails. In either case, the essential points are that the final cytoplasmic conformational change creates a site that attracts the initiating protein to the TCR complex and that each TCR has the potential to initiate some type of signal independent of aggregation [12],[14],[16].

Evidence for and against these models has accumulated over the years. The strongest evidence for the aggregation hypothesis has been that, in general, monovalent ligands in solution are ineffective in inducing TCR activation, whereas a wide variety of immobilized or soluble polyvalent ligands (multimerized anti-Vβ, anti-Cβ, and anti-CD3ε monoclonal antibodies, MHC presented bacterial and viral superantigens and, of course, specific MHC/peptide on APCs) all efficiently activate T cells. Furthermore, many microscopy studies have confirmed the presence of aggregated TCR complexes in activation clusters that form at the interface between T cells and APCs [8],[10],[11],[43],[45]. Recently, some studies have suggested that the minimal signaling unit on a T cell may be a cluster of just two TCR complexes [46],[47].

However, other data have been difficult to explain by a simple aggregation model and have led to proposals for some type of allosteric signaling. Initially, this idea arose from the demonstration that some anti-TCR antibodies and “altered peptide ligands” activated T cells poorly despite apparently reasonable TCR binding (reviewed in [17]). More recently, more direct evidence has been reported showing that the TCR engagement leads to the exposure of a proline-rich sequence (PRS) in the cytoplasmic tail of CD3ε, which then recruits the ubiquitously expressed adaptor protein Nck [14],[16]. This rapid change can happen at 0°C in the apparent absence of aggregation and is independent of any tyrosine kinase activity. The physiological importance of the PRS region, however, is still under debate. An earlier study showed that the mice without PRS region had normal T cell development and function [48]. A more recent study done by Mingueneau et al. [49], however, found that although the mature TCR transgenic T cells carrying the deletion of the PRS region responded to MHC/peptide normally, the DP thymocytes in such mice had up-regulated surface expression of the TCR complex. The study also showed that the PRS region enhanced TCR sensitivity to weak MHC/peptide ligands.

How such an allosteric signal might cross the membrane is still unknown. In one analysis of crystallographic data comparing TCRs in a bound versus free state, conformational adjustments at the interface between TCR and MHC/peptide were common, but there were no obvious conformational changes in Cα and Cβ regions upon ligand binding, with one exception (reviewed in [50]). However, more recently a similar analysis has concluded that there is a conformational change in Cα upon TCR engagement that is required for effective signal transduction [51], consistent with the idea of a direct intramolecular allosteric signal. Alternatively, several recent studies have argued for mechanical allosteric TCR signaling. For example, Sun et al. have suggested a piston-like signaling mechanism for the TCR, in which antigen engagement causes a mechanical force on the TCR complex pulling on the CD3 transmembrane regions changing their membrane proximal intracellular conformation [22]. Others have suggested a ligand induced mechanical displacement and/or reorientation of the CD3 heterodimers within the TCR complex [13],[18],[52]. A study done by Ma et al. [12] has suggested that such a mechanical force is generated by the TCR pulling away from MHC/peptide during the T cell/APC interaction.

Allosteric and aggregation mechanisms are not mutually exclusive and it may be that both contribute to the efficiency of TCR signaling. For example, Minguet et al. [18] have concluded that both TCR aggregation and exposure of the cytoplasmic Nck binding site were required for optimal T cell activation. They propose a new model of TCR activation called permissive geometry, in which the role of aggregation and conformational change are combined [53]. In this model, the existence of MHC/peptide dimers or multimers is required, because this leads to a reorientation of the subunits (TCRα, β, and CD3 ε, δ, and γ) within the TCR complex that is required for a full TCR activation.

Our results with mutated CD3ε argue against a simple aggregation model for TCR signaling (Figure 6B), since if bringing multiple CD3 cytoplasmic tails into close proximity were sufficient for signaling, we would not expect our finding of compromised signaling via the tgε^mut^ TCR even after very strong TCR cross-linking with a high affinity anti-Cβ or anti-CD3ε mAb. However, we cannot rule out that CXXC motif might still play a role in the correct orientation of the CD3ε cytoplasmic tails during aggregation.

Our ability to test allostery models via direct biochemical or cell biological approaches in our mice were limited by the small number of naïve mutant T cells we can isolate from our mice, e.g. not enough to perform Nck binding experiments ala the Alarcón group [16]. However, we wanted to attempt some experiments to relate our results to those of this group. Therefore, we have tried the alternate approach of cytoplasmic staining with the APA1.1 Mab, since the Alarcón group has reported that this Mab binds to the PRS on the CD3e cytoplasmic tail, which is exposed only after TCR triggering [19]. We cytoplasmically stained our wt and mutant naïve T cells with APA1.1 before and after maximal TCR cross-linking. All four of the T cell populations stained well with the antibody, but unfortunately there were no differences in the staining patterns before and after stimulation, nor any difference between the wt and mutant T cells (unpublished data). Thus, we found that the APA1.1 binding site appeared fully exposed before activation with either type of cell. The reasons for the differences between our results and those of the Alarcón group are not apparent. The answer may lie in the different cell types (e.g. human Jurkat tumor cells or COS cells versus normal mouse T cells). However, even though our experiments have not yet tested directly any of the alternative allostery models, one could nevertheless predict that disrupting the rigidity or stability of the CD3ε stalk region by mutation of the cysteines in the CXXC motif might interrupt the transmission of either type of allosteric signal through the stalks to the transmembrane regions (Figure 6B).

The most dramatic effect of the CD3ε mutation during T cell development in the transgenic mice was a severe bottleneck in the thymus at DN to DP transition, a process driven by the pre-TCR, in which the pre-TCR alpha (pTα) chain that lacks a V-region replaces the TCR chain. The molecular mechanisms of signals transmitted through the pre-TCR are not fully understood. CD3ε is an essential component of this receptor [24],[25], which is thought to signal constitutively without the need of a ligand [7]. A recent study done by Yamasaki et al. [54] showed that the pre-TCR complex can spontaneously form oligomers. Also, when the authors created a pre-formed chimeric dimer by fusing the transmembrane and extracellular regions of CD8 to the cytoplasmic region of CD3ε, they found that this molecule was sufficient to promote pre-TCR signaling, but did not activate peripheral T cells.

These results could mean that aggregation is all that is required for pre-TCR signaling and that the extracellular regions of the receptor components may be important for surface expression, but not for signaling. If this were the case, the drastic reduction in pre-T receptor mediated differentiation in our mutant mice could be due to poor incorporation of the mutant CD3ε into the pre-TCR complex, rather than an essential role for the CXXC motif in signal transduction. Unfortunately, we have not been able to answer this question, since the pre-TCR is normally expressed at very low levels and we have not yet succeeded in comparing quantitatively its surface expression on DN3 thymocytes from tgε^WT^ to tgε^Mut^ mice.

The effect of mutating the CXXC motif in CD3ε was much more dramatic on the thymic development of CD4^+^ T cells than of CD8^+^ T cells. The “instructive model” of CD4-CD8 lineage commitment in the thymus (reviewed in [55]) proposes that the signal strength/intensity initiated by the TCR-ligand interaction is one of the important “instructors” that determines the CD4/CD8 fate of a DP thymocyte. Thus, if a weak signal defaults the cell to the CD8 pathway, it is possible that the thymocytes with mutated CD3ε preferentially differentiated into CD8^+^ T cells, including those that would have normally differentiated into CD4^+^ T cells. This might also explain the changed Vβ and Vα repertoire in the periphery (Figure S1).

Finally, our studies point to the stalk region of CD3ε as important for TCR signal transduction, complementing the studies of Naeher et al. [56] on the importance of the stalk region connecting TCR Cα to its transmembrane region in T cell development and function. These results raise the question of whether in the fully assembled TCR complex the four CXXC containing stalks of the CD3 proteins (1γ, 1δ, and 2ε) combine with the TCR α and β stalk peptides to form a stable bundle that is essential for proper TCR signaling. Solving the atomic details of this complex has so far eluded structural biologists.

## Materials and Methods

### Animals

All animals were handled in strict accordance with good animal practice as defined by the relevant national and/or local animal welfare bodies, and all animal work was approved by the National Jewish Health Animal Care and Use Committee–Protocol N. AS2517.

C57BL/6J (BL6) mice were obtained from The Jackson Laboratory. cd3ε^−/−^ mice, which were originally obtained from The Jackson Laboratory as B6;129-CD3e^tm1Lov^/J mice [24], were backcrossed for more than seven generations to the BL6 strain and bred at National Jewish Health animal facility. Transgenic mice bearing the non-mutated stalk region of CD3ε (ε^WT^) or a variant with two C to S mutations (ε^Mut^) were generated in the same facility. Briefly, cd3ε cDNA was obtained using RT-PCRs from the splenocyte mRNA of normal BL6 mice. A multi-step PCR was performed to add a human c-Myc tag sequence “EQKLISEEDL” between the N terminus and signal peptide with a linker “GGGS” sequence and a flash tag sequence “CCPGCC” to the C terminus of CD3ε (Figure 1B). PCR products were cloned between two EcoRI restriction sites using a pCRII-TOPO TA cloning kit (TOPO TA Cloning®, Invitrogen). Point mutations then were introduced using PCRs (QuikChange II, Stratagene) to create mutated stalk regions carrying serines in place of the cysteines. All subcloned constructs were confirmed by sequencing. Then, nonmutated and mutated sequences were separately introduced into an improved human CD2 minigene cassette [57] using the EcoRI sites. Founder mice were generated by standard transgenic procedure using BL6 embryos. For each line, at least two founder mice were identified and crossed to cd3ε^−/−^ mice to provide F_2_ progeny. Those with a transgenic and endogenous cd3ε^−/−^ genotype (tgε^Mut or WT^cd3ε^−/−^, abbreviated as tgε^Mut^ or tgε^WT^) were monitored by cell surface expression of c-Myc (see Flow Cytometry for details) and competitive PCR assays.

### Primers

All primers were synthesized in the Molecular Resource Center of the National Jewish Medical and Research Center. Primers used for mutating the two conserved cysteines in the stalk region of mouse CD3ε were as follows: 5′-CTGAAAGCTCGAGTGTCCGAGTACTCCGTGGAGGTGGACC-3′ (forward primer) and 5′-GGTCCACCTCCACGGAGTACTCGGACACTCGAGCTTTCAG-3′ (reverse primer). Primers used for determining the presence of the endogenous cd3ε gene were as follows: 5′-TACAAAGTCTCCATCTCAGG-3′ (forward primer), 5′-CTCCTATTGTCTATTGTGATGC-3′ (reverse primer), and 5′-GACTGAATCTGTTTACTACTGAG-3′ (reverse primers).

### Antibodies

Anti-BrdU (Alexa Fluor 647 conjugated) antibodies were purchased from Invitrogen. Rabbit anti phospho-ERK antibodies were purchased from Cell Signaling Technology. Donkey anti rabbit antibodies (Alexa Fluor 647 conjugated IgG) were purchased from Invitrogen. Antibodies against B220 (Pacific Blue-conjugated RA3-6B2) were purchased from eBioscience. Antibodies against CD4 (APC-Cy7-conjugated L3T4), CD5 (FITC- or PE-conjugated 53–7.3), CD8α (APC- or PerCp-conjugated 53–6.7), CD25 (PE- or PE-Cy7-conjugated PC61), CD62L (PE- or PE-Cy7-conjugated MEL-14), CD69 (PE-conjugated H1.2F3), and Thy1.2 (FITC-conjugated 53–2.1) were purchased from BD Pharmingen. Antibodies against CD3ε (unconjugated or FITC-conjugated 145-2C11), CD44 (Alexa Fluor 488-conjugated IM7), c-Myc (biotin-conjugated 9E10), mouse Fc receptor (24G2), and TCR-Cβ (Alexa Fluor 647 or biotin-conjugated H57–597) were purified and/or coupled to the fluorochromes or biotin using standard methods in the Kappler/Marrack lab at the National Jewish Health.

### Flow Cytometry

Cell suspensions of mouse tail blood, spleen, lymph nodes (inguinal, brachial, and axillary), and thymus were prepared according to standard protocols, and stained with fluorescent or primary antibodies in BSS wash buffer (balanced salt solution with 0.5% FBS and 0.1% sodium azide) containing at least 20% 24G2 to block non-specific staining by Fc receptor. In some cases, cells were further stained with the appropriate secondary antibodies or APC-conjugated streptavidin, as described for the primary antibodies.

Analytical flow cytometry was performed with a Cyan flow cytometer (Dako Cytomation) and data were analyzed using FlowJo software (Tree Star, Inc.). Only live cells determined by forward and side scatter were used for analysis. A B220 dump channel was also included in some of the experiments to exclude nonspecific signals from other populations of cells (B cells, macrophage, etc.) found in the spleen, lymph nodes, and thymus of the transgenic mice.

To screen mice that expressed the transgenes, peripheral blood leukocytes were co-stained with antibodies against Thy1.2 and c-Myc. The live cells were first gated based on Thy1.2 expression. The Thy1.2^+^ population from the transgenic mice was then overlaid with the population from a BL6 mouse in terms of c-Myc staining (Figure S4). Only those mice with at least 2-fold increases of c-Myc staining were further analyzed.

### BrdU Incorporation Assay

For analysis of cell homeostatic proliferation using BrdU, mice were given drinking water containing 0.8 mg/ml BrdU (freshly made each day) for 9 consecutive days before sacrifice as previously described [30]. Cell preparation was carried out as described previously [58]. Finally, cells were stained with Alexa Fluor 647-coupled anti-BrdU antibodies on ice for 30 min, washed, and resuspended in PBS wash buffer for flow cytometry analysis.

### TCR Cross-Linking and T Cell Activation Assays

The day before the experiment, the wells of a 96-well flat bottom plate were coated with 1) mAbs against TCR-Cβ (H57-597) at a concentration of 10 µg/well, 2) mAbs against CD3ε (145-2C11) at a concentration of 10 µg/well, or 3) ExtrAvidin (Sigma) at a concentration of 7.5 µg/well at 4°C overnight. The next day, the plate was washed and blocked with CTM (complete tumor medium, MEM enriched with essential and nonessential amino acids, glutamine, sodium bicarbonate, 10% fetal bovine serum, and 20 µM 2-mercaptoethanol) at room temperature until it was used.

To generate samples whose TCRs were occupied with a different amount of anti Cβ mAbs, T cells from nylon wool-purified spleen and lymph nodes cells were first seeded in duplicate in the wells of another uncoated 96-well plate at 1 million cells per well. They were then incubated with a biotin-conjugated anti-TCR Cβ mAb (H57–597) at various concentrations, in the presence of the anti-Fc receptor Mab, 24G2, on ice for 30 min. After the treatment, unbound antibodies were washed away. One of the duplicates was stained with APC-conjugated streptavidin (BD Pharmingen) and analyzed using flow cytometry to determine the level of TCR occupancy. The other duplicate was transferred on ice to the wells of ExtrAvidin-coated plate at a final concentration of less than 2.5×10^5^ cells/well for receptor cross-linking.

Alternatively, nylon wool-purified T cells were directly seeded in the plates coated with mAbs. All types of transferred cells were then briefly spun down to the bottom (500 × g at 0°C for 2 min) to achieve rapid and maximal activation, and incubated at 37°C with CO_2_ for various time points. In some experiments, total splenocytes were seeded in an untreated plate at 5×10^6^ cells/well and incubated with SEB at a final concentration of 10 µg/ml in CTM.

For studying intracellularly phosphorylated ERK, after 20 min stimulation, paraformaldehyde (Sigma) solution was added to the wells at a final concentration of 2% to stop the reaction. The phospho-ERK staining was then performed according to the manufacturer's protocol. The samples were then stained with Alexa Fluor 647-coupled anti-rabbit antibodies, and were then analyzed using flow cytometry.

For studying induced surface CD69, cells after at least 8 h stimulation were harvested and washed in BSS wash buffer. Samples then were stained with antibodies against CD69 as well as other surface markers, including B220, CD4, CD8, CD44, and TCR-Vβs, according to routine protocol for multi-color flow cytometry.

### Graphs and Statistics

Graphs of numerical data and statistics were generated using Prism 4 program (GraphPad Software, Inc.).

### Online Supplemental Material

Four figures are included in the supplemental material. Figure S1 compares the Vα and Vβ repertoires in the tgε^Mut^ and tgε^WT^ mice. Figure S2 shows the differences in pERK and CD69 induction in total peripheral T cells from tgε^Mut^ and tgε^Wt^ mice after TCR cross-linking. Figure S3 shows the competition in a T cell hybridoma between endogenous and transduced CD3ε for incorporation into the surface expressed TCR complex. Figure S4 shows the histograms of surface expressions of CD3ε transgenes in the backcrossed endogenous-cd3ε-null transgenic mice (tgε^WT^ or tgε^Mut^) by staining c-Myc tag on the cell surface.

## Supporting Information

Figure S1 **Transgenic mice bearing cysteine-mutated CD3ε had a changed but not oligoclonal T cell repertoire.** Vα usage in CD4^+^ (A) or CD8^+^ (B), and Vβ usage in CD4^+^ (C) or CD8^+^ (D) T cells were determined by flow cytometry. Cells from spleens were costained with anti-B220, anti-CD4, anti-CD8, and different anti-TCR V segment antibodies. The use of different V chains was calculated by gating on the CD4^+^ or CD8^+^ cells from the alive and B220- population. “Cntl” refers to combined wt control mice as described in Figure 2. Data shown are for 4 tgε^Mut^ mice and 7 wt control mice (3 BL6, 3 tgε^Mut^cd3ε^+/−^, and 1 tgε^WT^). Data are indicated as the mean ± SEM. *p* values were obtained from *t* tests.
(0.16 MB PDF)

Figure S2 **Peripheral total T cells from tgεMut mice did not respond properly after cross-linking the TCRs.** (A, B) Curves for phospho-ERK1/2 against the degree of TCR occupancy in total CD4^+^ or CD8^+^ T cell populations. T cells were from nylon wool-purified spleen and lymph node (inguinal, brachial, and axillary) cells. The data were acquired as described in Figure 5C, except total CD4^+^ or CD8^+^ T cells were assessed. The data represent two independent experiments. In each experiment, cells of each group were pooled from two individual animals. (C) CD69 induction on total spleen T cells after TCR cross-linking. Splenocytes from different mice were stimulated by plate-bound anti-TCRβ and anti-CD28 antibodies at 37°C for 17 h. After the stimulation, cells were harvested and CD69 induction was determined by flow cytometry. Data shown are for 2 tgε^Mut^ mice and 5 wt control mice (1 BL6, 1 tgεWTcd3ε+/−, 1 tgεMutcd3ε+/−, and 2 tgε^WT^). “Cntl” refers to the combined wt control mice as described in Figure 2. The data are shown as the mean ± SEM. The *p* value was obtained from a *t* test. The data represent two independent experiments.
(0.04 MB PDF)

Figure S3 **Various expression levels of the mutant and wt proteins from CD3ε constructs were detected on the cell surface, which was not due to the variation in transduction or protein translation.** (A) Histograms of GFP or c-Myc staining from vector only (MiG, grey filled), c-Myc tagged wt CD3ε (ε^WT^, solid line), or mutated CD3ε (ε^Mut^, dashed line) retrovirally-transduced T hybridoma cells. DNA fragments of non-mutated or mutated CD3ε with tag sequences (Figure 1B) were cloned into a mouse MSCV retrovirus expression vector which independently expressed GFP (MiG). The constructs were used to transduce B3K0508 T hybridoma cells. Transduced cells were first sorted based on GFP expression. GFP-positive cells were then cultured after sorting and stained with antibodies against the c-Myc tag. The left panel is the overlay of GFP levels gated from live cells. The right panel is the overlay of c-Myc levels gated from both live and GFP-positive cells. (B) Western blots of the whole cell lysates from CD3ε transduced hybridoma cells. Various B3K0508 cells (2 million each) that were transduced with different CD3ε constructs were directly lysed in SDS-PAGE buffer. Samples were immunoblotted with anti-CD3ε antibodies. “CD3ε-Trans” refers to transduced CD3ε proteins. “CD3ε-Endo” refers to endogenous CD3ε proteins. The result is representative of more than three independent experiments.
(0.05 MB PDF)

Figure S4 **Histograms of staining for c-Myc expressed on the cell surface from the endogenous-CD3ε-null transgenic mice (tgε^WT^ or tgε^Mut^).** PBLs from individual mice with these genotypes were costained with anti-Thy 1.2 and anti-c-Myc antibodies. The Thy1.2+ population from the transgenic mice (red line) was overlaid with the Thy1.2^+^ population from a BL6 mouse (grey filled). The mean fluorescent intensity (MFI) values are indicated.
(0.03 MB PDF)

## Abbreviations

APCs: antigen presenting cells
BrdU: bromodeoxyuridine
CTM: complete tumor medium
DN: CD4^−^CD8^−^
DP: CD4^+^CD8^+^
SP: CD4^+^CD8^−^ or CD4^−^CD8^+^
MHC: major histocompatibility complex
MFI: mean fluorescence intensity
PRS: proline-rich sequence
TCR: T cell receptor
wt: wild-type

## References

[1] Malissen B, Ardouin L, Lin S. Y, Gillet A, Malissen M (1999). Function of the CD3 subunits of the pre-TCR and TCR complexes during T cell development.. Adv Immunol.

[2] Clevers H, Alarcon B, Wileman T, Terhorst C (1988). The T cell receptor/CD3 complex: a dynamic protein ensemble.. Annu Rev Immunol.

[3] Alarcon B, Berkhout B, Breitmeyer J, Terhorst C (1988). Assembly of the human T cell receptor-CD3 complex takes place in the endoplasmic reticulum and involves intermediary complexes between the CD3-gamma.delta.epsilon core and single T cell receptor alpha or beta chains.. J Biol Chem.

[4] San Jose E, Sahuquillo A. G, Bragado R, Alarcon B (1998). Assembly of the TCR/CD3 complex: CD3 epsilon/delta and CD3 epsilon/gamma dimers associate indistinctly with both TCR alpha and TCR beta chains. Evidence for a double TCR heterodimer model.. Eur J Immunol.

[5] Sancho J, Chatila T, Wong R. C, Hall C, Blumberg R (1989). T-cell antigen receptor (TCR)-alpha/beta heterodimer formation is a prerequisite for association of CD3-zeta 2 into functionally competent TCR.CD3 complexes.. J Biol Chem.

[6] Minami Y, Weissman A. M, Samelson L. E, Klausner R. D (1987). Building a multichain receptor: synthesis, degradation, and assembly of the T-cell antigen receptor.. Proc Natl Acad Sci U S A.

[7] von Boehmer H, Fehling H. J (1997). Structure and function of the pre-T cell receptor.. Annu Rev Immunol.

[8] Varma R, Campi G, Yokosuka T, Saito T, Dustin M. L (2006). T cell receptor-proximal signals are sustained in peripheral microclusters and terminated in the central supramolecular activation cluster.. Immunity.

[9] Barcia C, Thomas C. E, Curtin J. F, King G. D, Wawrowsky K (2006). In vivo mature immunological synapses forming SMACs mediate clearance of virally infected astrocytes from the brain.. J Exp Med.

[10] Grakoui A, Bromley S. K, Sumen C, Davis M. M, Shaw A. S (1999). The immunological synapse: a molecular machine controlling T cell activation.. Science.

[11] Monks C. R, Freiberg B. A, Kupfer H, Sciaky N, Kupfer A (1998). Three-dimensional segregation of supramolecular activation clusters in T cells.. Nature.

[12] Ma Z, Sharp K. A, Janmey P. A, Finkel T. H (2008). Surface-anchored monomeric agonist pMHCs alone trigger TCR with high sensitivity.. PLoS Biol.

[13] Kuhns M. S, Davis M. M (2007). Disruption of extracellular interactions impairs T cell receptor-CD3 complex stability and signaling.. Immunity.

[14] Gil D, Schrum A. G, Alarcon B, Palmer E (2005). T cell receptor engagement by peptide-MHC ligands induces a conformational change in the CD3 complex of thymocytes.. J Exp Med.

[15] Kjer-Nielsen L, Clements C. S, Purcell A. W, Brooks A. G, Whisstock J. C (2003). A structural basis for the selection of dominant alphabeta T cell receptors in antiviral immunity.. Immunity.

[16] Gil D, Schamel W. W, Montoya M, Sanchez-Madrid F, Alarcon B (2002). Recruitment of Nck by CD3 epsilon reveals a ligand-induced conformational change essential for T cell receptor signaling and synapse formation.. Cell.

[17] Janeway C. A (1995). Ligands for the T-cell receptor: hard times for avidity models.. Immunol Today.

[18] Minguet S, Swamy M, Alarcon B, Luescher I. F, Schamel W. W (2007). Full activation of the T cell receptor requires both clustering and conformational changes at CD3.. Immunity.

[19] Risueno R. M, Gil D, Fernandez E, Sanchez-Madrid F, Alarcon B (2005). Ligand-induced conformational change in the T-cell receptor associated with productive immune synapses.. Blood.

[20] Xu C, Call M. E, Wucherpfennig K. W (2006). A membrane-proximal tetracysteine motif contributes to assembly of CD3deltaepsilon and CD3gammaepsilon dimers with the T cell receptor.. J Biol Chem.

[21] Thomassen E. A, Dekking E. H, Thompson A, Franken K. L, Sanal O (2006). The impact of single amino acid substitutions in CD3gamma on the CD3epsilongamma interaction and T-cell receptor-CD3 complex formation.. Hum Immunol.

[22] Sun Z. J, Kim K. S, Wagner G, Reinherz E. L (2001). Mechanisms contributing to T cell receptor signaling and assembly revealed by the solution structure of an ectodomain fragment of the CD3 epsilon gamma heterodimer.. Cell.

[23] Borroto A, Mallabiabarrena A, Albar J. P, Martinez A. C, Alarcon B (1998). Characterization of the region involved in CD3 pairwise interactions within the T cell receptor complex.. J Biol Chem.

[24] DeJarnette J. B, Sommers C. L, Huang K, Woodside K. J, Emmons R (1998). Specific requirement for CD3epsilon in T cell development.. Proc Natl Acad Sci U S A.

[25] Malissen M, Gillet A, Ardouin L, Bouvier G, Trucy J (1995). Altered T cell development in mice with a targeted mutation of the CD3-epsilon gene.. Embo J.

[26] Azzam H. S, Grinberg A, Lui K, Shen H, Shores E. W (1998). CD5 expression is developmentally regulated by T cell receptor (TCR) signals and TCR avidity.. J Exp Med.

[27] Yamashita I, Nagata T, Tada T, Nakayama T (1993). CD69 cell surface expression identifies developing thymocytes which audition for T cell antigen receptor-mediated positive selection.. Int Immunol.

[28] Swat W, Dessing M, von Boehmer H, Kisielow P (1993). CD69 expression during selection and maturation of CD4+8+ thymocytes.. Eur J Immunol.

[29] Bendelac A, Matzinger P, Seder R. A, Paul W. E, Schwartz R. H (1992). Activation events during thymic selection.. J Exp Med.

[30] Tough D. F, Sprent J (1994). Turnover of naive- and memory-phenotype T cells.. J Exp Med.

[31] Tanchot C, Le Campion A, Leaument S, Dautigny N, Lucas B (2001). Naive CD4(+) lymphocytes convert to anergic or memory-like cells in T cell-deprived recipients.. Eur J Immunol.

[32] Goldrath A. W, Bogatzki L. Y, Bevan M. J (2000). Naive T cells transiently acquire a memory-like phenotype during homeostasis-driven proliferation.. J Exp Med.

[33] Cho B. K, Rao V. P, Ge Q, Eisen H. N, Chen J (2000). Homeostasis-stimulated proliferation drives naive T cells to differentiate directly into memory T cells.. J Exp Med.

[34] Kieper W. C, Jameson S. C (1999). Homeostatic expansion and phenotypic conversion of naive T cells in response to self peptide/MHC ligands.. Proc Natl Acad Sci U S A.

[35] Tanchot C, Lemonnier F. A, Perarnau B, Freitas A. A, Rocha B (1997). Differential requirements for survival and proliferation of CD8 naive or memory T cells.. Science.

[36] Takeda S, Rodewald H. R, Arakawa H, Bluethmann H, Shimizu T (1996). MHC class II molecules are not required for survival of newly generated CD4+ T cells, but affect their long-term life span.. Immunity.

[37] Whitehurst C. E, Boulton T. G, Cobb M. H, Geppert T. D (1992). Extracellular signal-regulated kinases in T cells. Anti-CD3 and 4 beta-phorbol 12-myristate 13-acetate-induced phosphorylation and activation.. J Immunol.

[38] Testi R, Phillips J. H, Lanier L. L (1989). Leu 23 induction as an early marker of functional CD3/T cell antigen receptor triggering. Requirement for receptor cross-linking, prolonged elevation of intracellular [Ca++] and stimulation of protein kinase C.. J Immunol.

[39] White J, Herman A, Pullen A. M, Kubo R, Kappler J. W (1989). The V beta-specific superantigen staphylococcal enterotoxin B: stimulation of mature T cells and clonal deletion in neonatal mice.. Cell.

[40] Kappler J, Kozono H, Clements J, Crawford F, White J (1997). Interactions of T-cell receptor V elements with staphylococcal superantigens; 1997. MUNKSGAARD BOGHANDEL.

[41] Blumberg R. S, Ley S, Sancho J, Lonberg N, Lacy E (1990). Structure of the T-cell antigen receptor: evidence for two CD3 epsilon subunits in the T-cell receptor-CD3 complex.. Proc Natl Acad Sci U S A.

[42] Fewtrell C, Metzger H (1980). The role of aggregation in the function of the IgE Fc receptors.. J Reticuloendothel Soc.

[43] Yokosuka T, Sakata-Sogawa K, Kobayashi W, Hiroshima M, Hashimoto-Tane A (2005). Newly generated T cell receptor microclusters initiate and sustain T cell activation by recruitment of Zap70 and SLP-76.. Nat Immunol.

[44] Ottemann K. M, Xiao W, Shin Y. K, Koshland D. E (1999). A piston model for transmembrane signaling of the aspartate receptor.. Science.

[45] Potter T. A, Grebe K, Freiberg B, Kupfer A (2001). Formation of supramolecular activation clusters on fresh ex vivo CD8+ T cells after engagement of the T cell antigen receptor and CD8 by antigen-presenting cells.. Proc Natl Acad Sci U S A.

[46] Irvine D. J, Purbhoo M. A, Krogsgaard M, Davis M. M (2002). Direct observation of ligand recognition by T cells.. Nature.

[47] Krogsgaard M, Li Q. J, Sumen C, Huppa J. B, Huse M (2005). Agonist/endogenous peptide-MHC heterodimers drive T cell activation and sensitivity.. Nature.

[48] Szymczak A. L, Workman C. J, Gil D, Dilioglou S, Vignali K. M (2005). The CD3epsilon proline-rich sequence, and its interaction with Nck, is not required for T cell development and function.. J Immunol.

[49] Mingueneau M, Sansoni A, Gregoire C, Roncagalli R, Aguado E (2008). The proline-rich sequence of CD3[epsi] controls T cell antigen receptor expression on and signaling potency in preselection CD4+CD8+ thymocytes.. Nat Immunol.

[50] Rudolph M. G, Stanfield R. L, Wilson I. A (2006). How TCRs bind MHCs, peptides, and coreceptors.. Annu Rev Immunol.

[51] Beddoe T, Chen Z, Clements C. S, Ely L. K, Bushell S. R (2009). Antigen ligation triggers a conformational change within the constant domain of the alphabeta T cell receptor.. Immunity.

[52] Kim S. T, Takeuchi K, Sun Z. Y, Touma M, Castro C. E (2009). The alphabeta T cell receptor is an anisotropic mechanosensor.. J Biol Chem.

[53] Minguet S, Schamel W. W (2008). A permissive geometry model for TCR-CD3 activation.. Trends Biochem Sci.

[54] Yamasaki S, Ishikawa E, Sakuma M, Ogata K, Sakata-Sogawa K (2006). Mechanistic basis of pre-T cell receptor-mediated autonomous signaling critical for thymocyte development.. Nat Immunol.

[55] Kappes D. J, He X, He X (2005). CD4-CD8 lineage commitment: an inside view.. Nat Immunol.

[56] Naeher D, Luescher I. F, Palmer E (2002). A role for the alpha-chain connecting peptide motif in mediating TCR-CD8 cooperation.. J Immunol.

[57] Zhumabekov T, Corbella P, Tolaini M, Kioussis D (1995). Improved version of a human CD2 minigene based vector for T cell-specific expression in transgenic mice.. J Immunol Methods.

[58] Lenz D. C, Kurz S. K, Lemmens E, Schoenberger S. P, Sprent J (2004). IL-7 regulates basal homeostatic proliferation of antiviral CD4+T cell memory.. Proc Natl Acad Sci U S A.

