# Supplementary figures and images for: A Conserved CXXC Motif in CD3ε Is Critical for T Cell Development and TCR Signaling
Source: PLoS Biol. 2009 Dec 1;7(12):e1000253. doi: 10.1371/journal.pbio.1000253 (PMC2776832; doi:10.1371/journal.pbio.1000253)

**A.**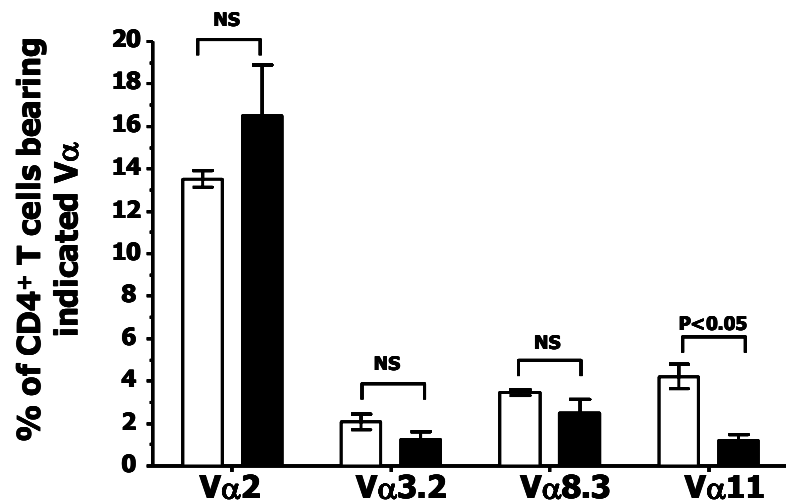**B.**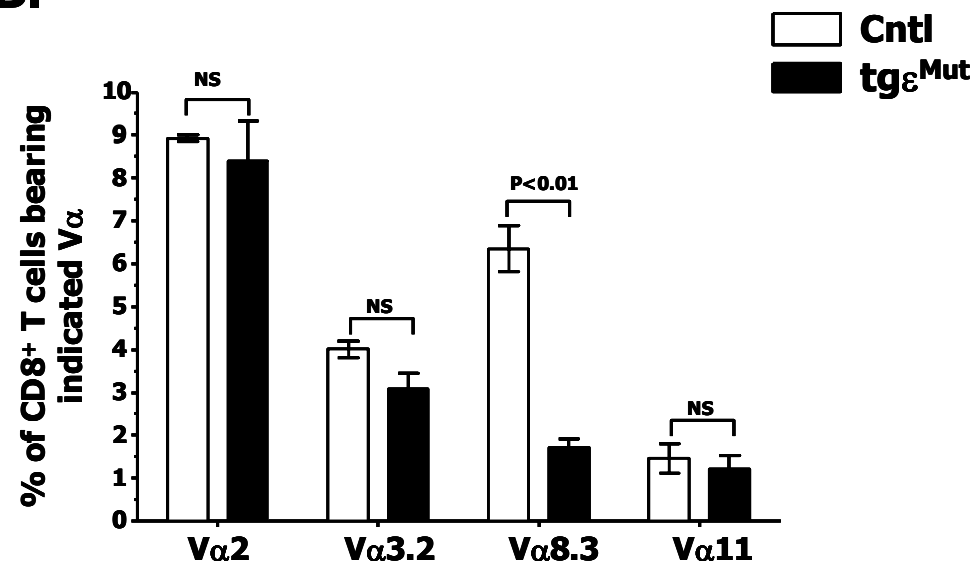**C.**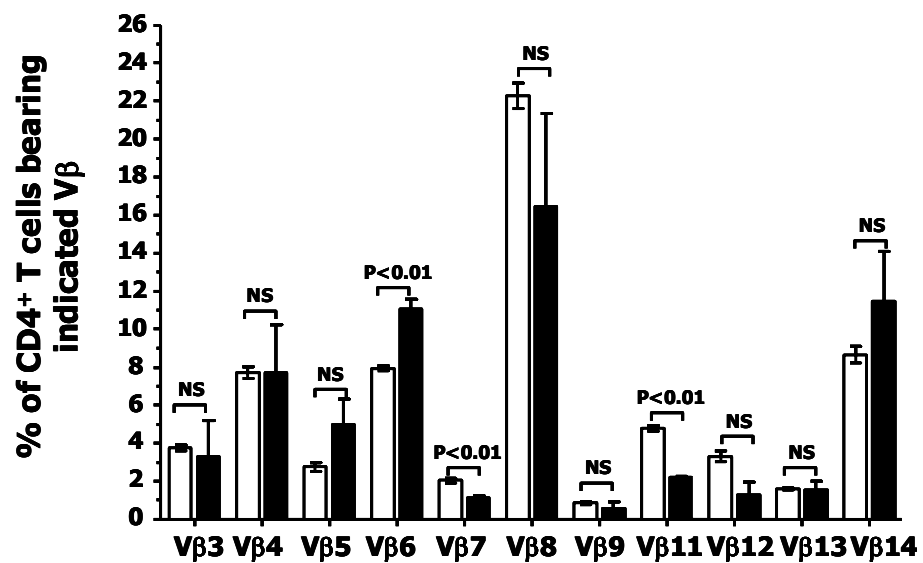**D.**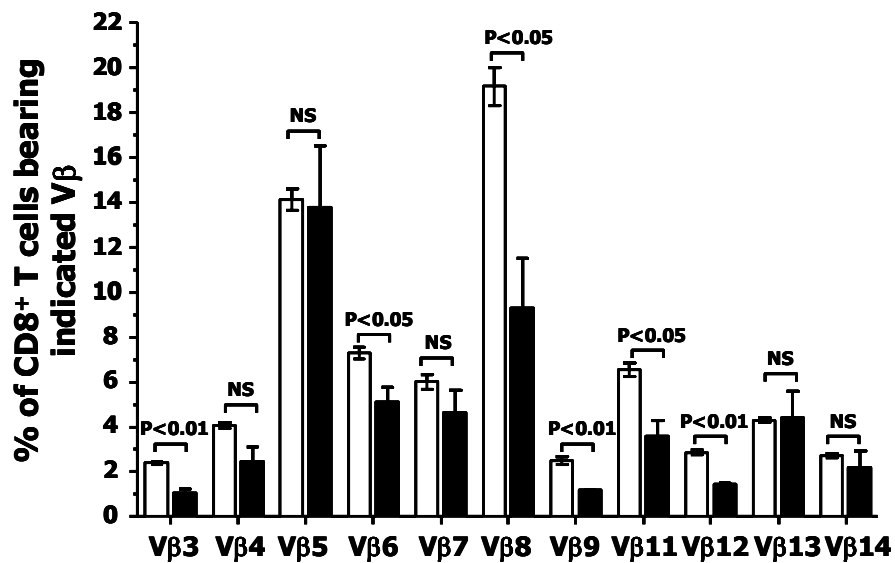

Supplement: Figure S1 — Transgenic mice bearing cysteine-mutated CD3ε had a changed but not oligoclonal T cell repertoire. Vα usage in CD4+ (A) or CD8+ (B), and Vβ usage in CD4+ (C) or CD8+ (D) T cells were determined by flow cytometry. Cells from spleens were costained with anti-B220, anti-CD4, anti-CD8, and different anti-TCR V segment antibodies. The use of different V chains was calculated by gating on the CD4+ or CD8+ cells from the alive and B220- population. “Cntl” refers to combined wt control mice as described in Figure 2. Data shown are for 4 tgεMut mice and 7 wt control mice (3 BL6, 3 tgεMutcd3ε+/−, and 1 tgεWT). Data are indicated as the mean ± SEM. p values were obtained from t tests. (0.16 MB PDF) [file pbio.1000253.s001.pdf]

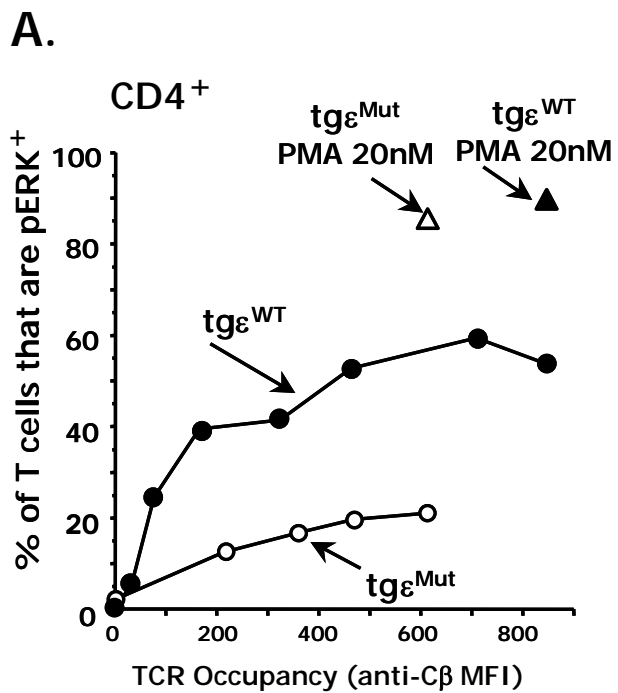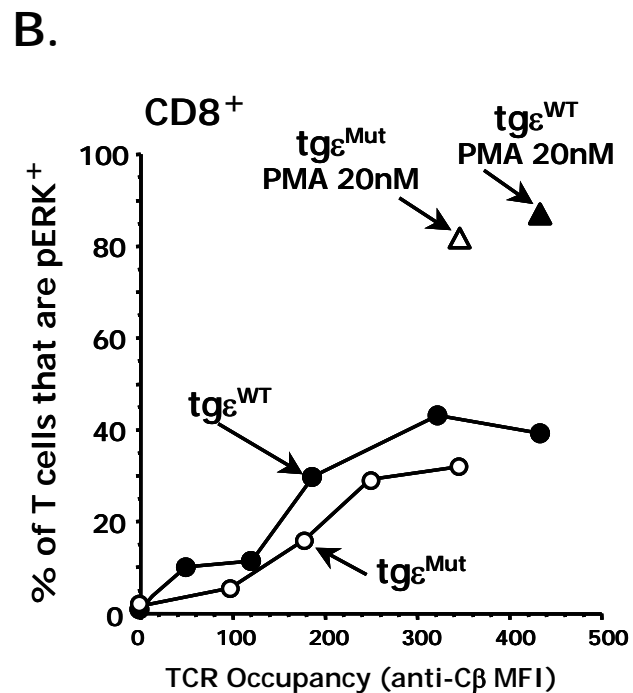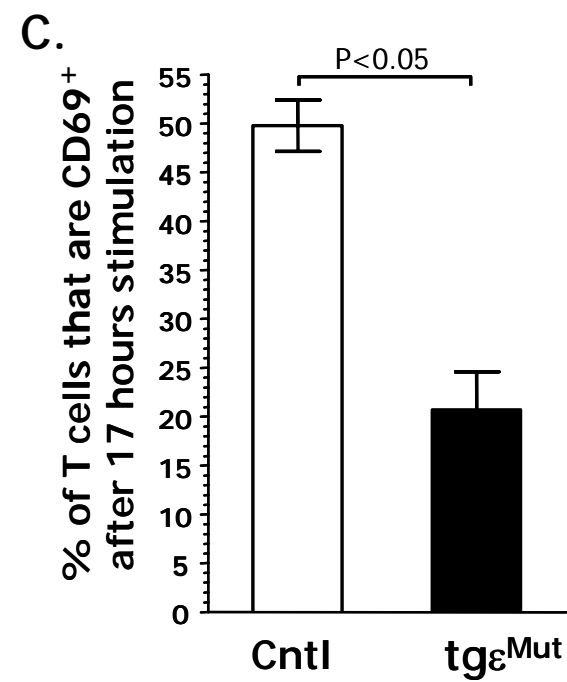

Supplement: Figure S2 — Peripheral total T cells from tgεMut mice did not respond properly after cross-linking the TCRs. (A, B) Curves for phospho-ERK1/2 against the degree of TCR occupancy in total CD4+ or CD8+ T cell populations. T cells were from nylon wool-purified spleen and lymph node (inguinal, brachial, and axillary) cells. The data were acquired as described in Figure 5C, except total CD4+ or CD8+ T cells were assessed. The data represent two independent experiments. In each experiment, cells of each group were pooled from two individual animals. (C) CD69 induction on total spleen T cells after TCR cross-linking. Splenocytes from different mice were stimulated by plate-bound anti-TCRβ and anti-CD28 antibodies at 37°C for 17 h. After the stimulation, cells were harvested and CD69 induction was determined by flow cytometry. Data shown are for 2 tgεMut mice and 5 wt control mice (1 BL6, 1 tgεWTcd3ε+/−, 1 tgεMutcd3ε+/−, and 2 tgεWT). “Cntl” refers to the combined wt control mice as described in Figure 2. The data are shown as the mean ± SEM. The p value was obtained from a t test. The data represent two independent experiments. (0.04 MB PDF) [file pbio.1000253.s002.pdf]

**A.**

Transduced T hybridoma cell line cells

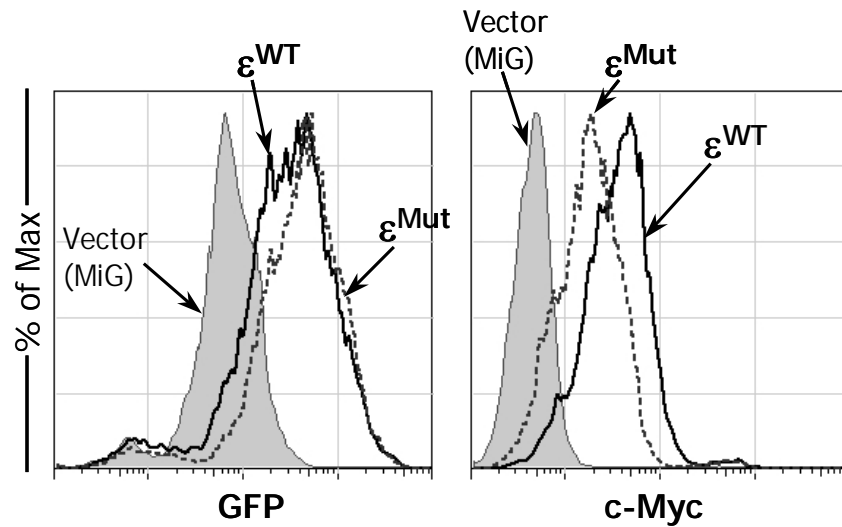

**B.**

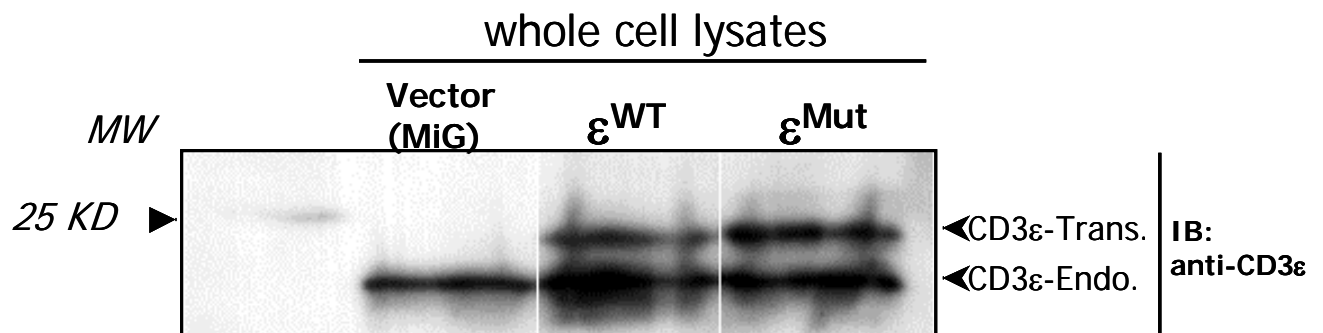

Supplement: Figure S3 — Various expression levels of the mutant and wt proteins from CD3ε constructs were detected on the cell surface, which was not due to the variation in transduction or protein translation. (A) Histograms of GFP or c-Myc staining from vector only (MiG, grey filled), c-Myc tagged wt CD3ε (εWT, solid line), or mutated CD3ε (εMut, dashed line) retrovirally-transduced T hybridoma cells. DNA fragments of non-mutated or mutated CD3ε with tag sequences (Figure 1B) were cloned into a mouse MSCV retrovirus expression vector which independently expressed GFP (MiG). The constructs were used to transduce B3K0508 T hybridoma cells. Transduced cells were first sorted based on GFP expression. GFP-positive cells were then cultured after sorting and stained with antibodies against the c-Myc tag. The left panel is the overlay of GFP levels gated from live cells. The right panel is the overlay of c-Myc levels gated from both live and GFP-positive cells. (B) Western blots of the whole cell lysates from CD3ε transduced hybridoma cells. Various B3K0508 cells (2 million each) that were transduced with different CD3ε constructs were directly lysed in SDS-PAGE buffer. Samples were immunoblotted with anti-CD3ε antibodies. “CD3ε-Trans” refers to transduced CD3ε proteins. “CD3ε-Endo” refers to endogenous CD3ε proteins. The result is representative of more than three independent experiments. (0.05 MB PDF) [file pbio.1000253.s003.pdf]

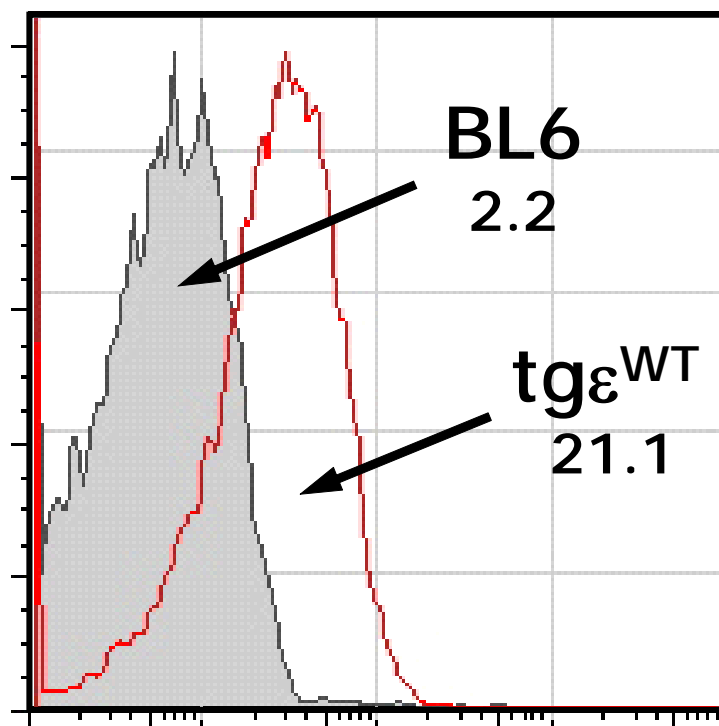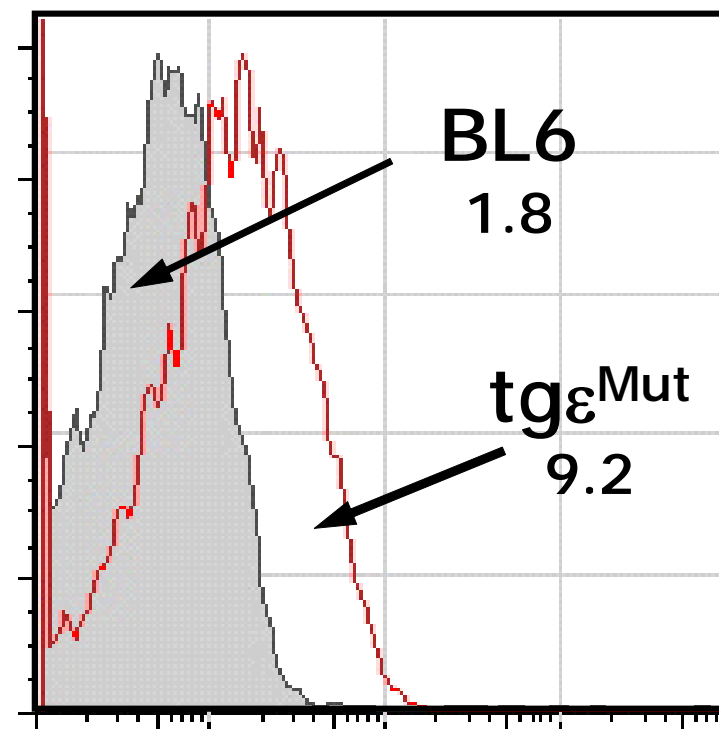

c-Myc →

Supplement: Figure S4 — Histograms of staining for c-Myc expressed on the cell surface from the endogenous-CD3ε-null transgenic mice (tgεWT or tgεMut). PBLs from individual mice with these genotypes were costained with anti-Thy 1.2 and anti-c-Myc antibodies. The Thy1.2+ population from the transgenic mice (red line) was overlaid with the Thy1.2+ population from a BL6 mouse (grey filled). The mean fluorescent intensity (MFI) values are indicated. (0.03 MB PDF) [file pbio.1000253.s004.pdf]
